# Graphie: A network-based visual interface for the UK's primary legislation

**DOI:** 10.12688/f1000research.129632.1

**Published:** 2023-03-03

**Authors:** Evan Tzanis, Pierpaolo Vivo, Yanik-Pascal Förster, Luca Gamberi, Alessia Annibale

**Affiliations:** 1 Quantitative and Digital Law Lab, Department of Mathematics, King's College London, London, WC2R 2LS, UK

**Keywords:** legal data science, legislation, data pipelines, network interfaces, visualization of legal texts, user interface

## Abstract

**Background:** legislation.gov.uk is a platform that enables users to explore and navigate the many sections of the UK’s legal corpus through its well-designed searching and browsing features. However, there is room for improvement as it lacks the ability to easily move between related sections or Acts and only presents a text-only rendering of provisions. With Graphie, our novel navigational tool (graphie.quantlaw.co.uk), we aim to address this limitation by presenting alternative visualizations of legal documents using both text and graphs.

**Methods: **The building block of Graphie is Sofia, an offline data pipeline designed to support different data visualizations by parsing and modelling data provided by legislation.gov.uk in open access form.

**Results: **Graphie provides a network representation of the hierarchical structure of an Act of Parliament, which is typically organized in a tree-like fashion according to the content and information contained in each sub-branch. Nodes in Graphie represent sections of an Act (or individual provisions), while links embody the hierarchical connections between them. The legal map provided by Graphie is easily navigable by hovering on nodes, which are also color-coded and numbered to provide easily accessible information about the underlying content. The full textual content of each node is also available on a dedicated hyperlinked canvas.

**Conclusions:** While we focus on the Housing Act 2004 for illustrative purposes, our platform is scalable, versatile, and provides users with a unified toolbox to visualize and explore the UK legal corpus in a fast and user-friendly way.

## 1. Introduction

The volume of the UK’s primary legislation keeps growing at a very fast pace. According to a rough estimate there are at least 176,890 [ 1] Public and General Acts currently in force in the UK – the exact number is not known – and an average of 30 new Public Acts are produced every year. ^1^ New legislation [ 2] documents are regularly uploaded to the UK’s Legislation web platform ( legislation.gov.uk ^2^), managed by The National Archives [ 3] (TNA) on behalf of HM Government.

**Figure 1.**
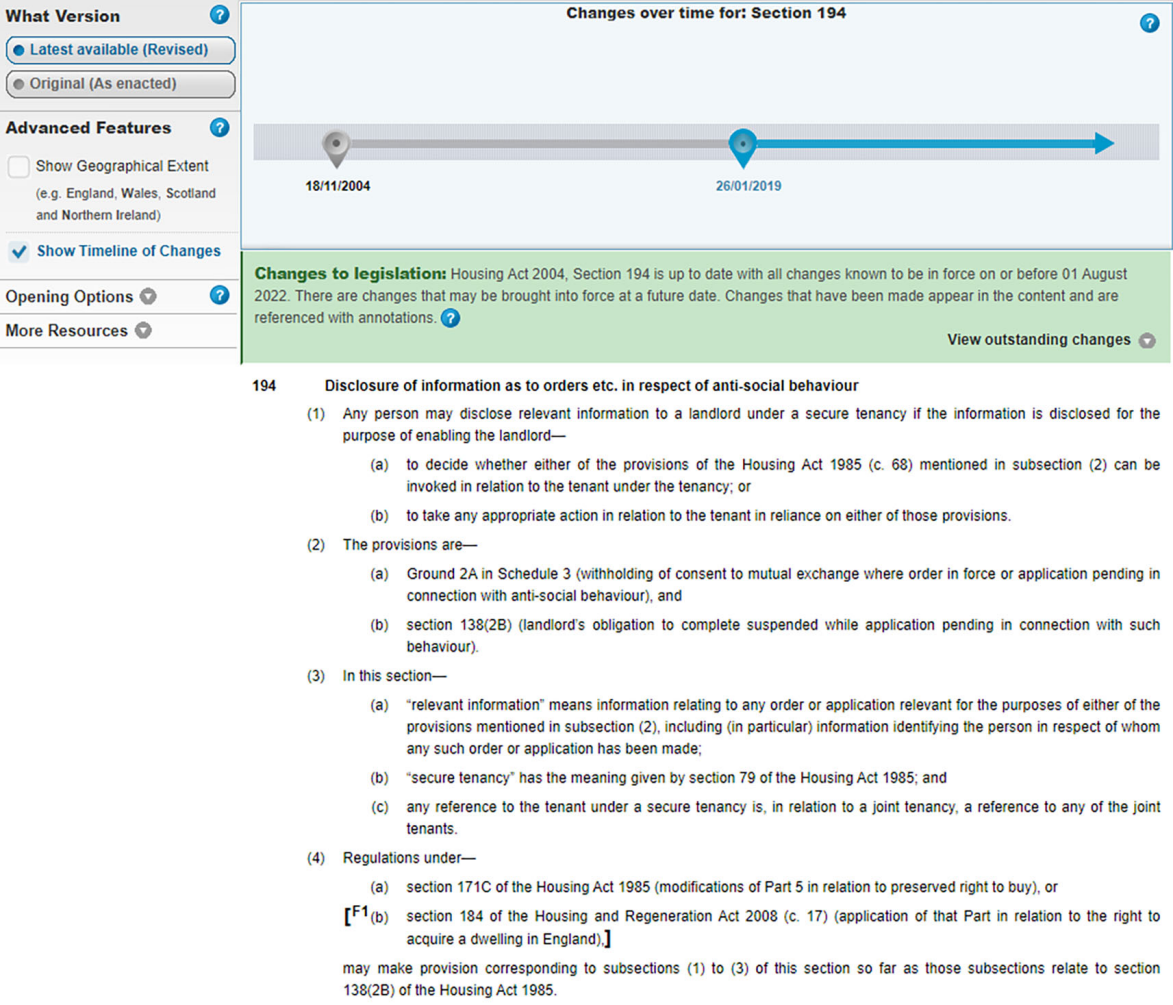
Section 194 of the Housing Act 2004, as provided by Ref. 2. The default visualization offering includes (i) plain text of the provision (with limited hyperlinking); (ii) a time-slider to access different versions of the provision; (iii), a choice between the latest version, or version as originally enacted.

Users may reach the legislation.gov.uk webpage while looking for a specific Act or provision on standard search engines. Others may use the platform as part of their daily job. The legislation.gov.uk website has been carefully designed and is maintained to cater for the needs of a diverse pool of stakeholders. It is built on clear principles and offers a number of essential features: first, users can keyword-query the database, and are offered an easy-to-use set of navigational links for browsing through different corners of the UK legislation. Secondly, legislation data are open-source and fully accessible via an API [ 4]. All API legal documents are held in XML format under a well-defined and concise set of persistent URIs [ 5]. Thanks to this API technology and to TNA’s open-access philosophy, the legislation data can also be connected and streamlined across other data sets and applications, such as for instance Westlaw, ^3^ a leading commercial legal research platform. In addition, the legislation.gov.uk platform enables users to enjoy the textual version of a whole Act – or a section/paragraph thereof – in HTML or in PDF formats. Acts are made available in both their original (as enacted) or revised (current) versions, and for those Acts with revisions, a detailed timeline highlighting any editing changes to legal documents over time is also provided (see Figure 1).

The standard set by TNA in terms of offering a digital and navigable version of essentially the entire corpus of UK legislation is high and very competitive on the world stage. However, the lack of easy “hopping” capabilities between items and provisions that should be naturally linked together, as well as its focus on a text-only rendering of provisions leaves room for some improvement.

As for academic papers, reading and understanding legislation requires concentration and time, and the ability to efficiently “follow the leads” between different provisions of the same Act – or between different Acts that have a bearing on the same matter. Consider again section 194 of the Housing Act 2004 as our main example, which is highly connected [ 6] with other sections from different Acts of the UK’s Statute Book. To fully understand the content and implications of section 194, the reader is expected to visit and read the sections of these other statutes referenced there first, and then hop onto the sections/provisions that these other sections might refer to, and to repeat this hopping routine exhaustively, covering all possible linkages between sections/provisions/statutes. Using a text-based visualization interface with limited hyperlinking capabilities such as that provided by Ref. 2 makes these tasks time-consuming and inefficient for long and highly interconnected sections.

**Figure 2.**
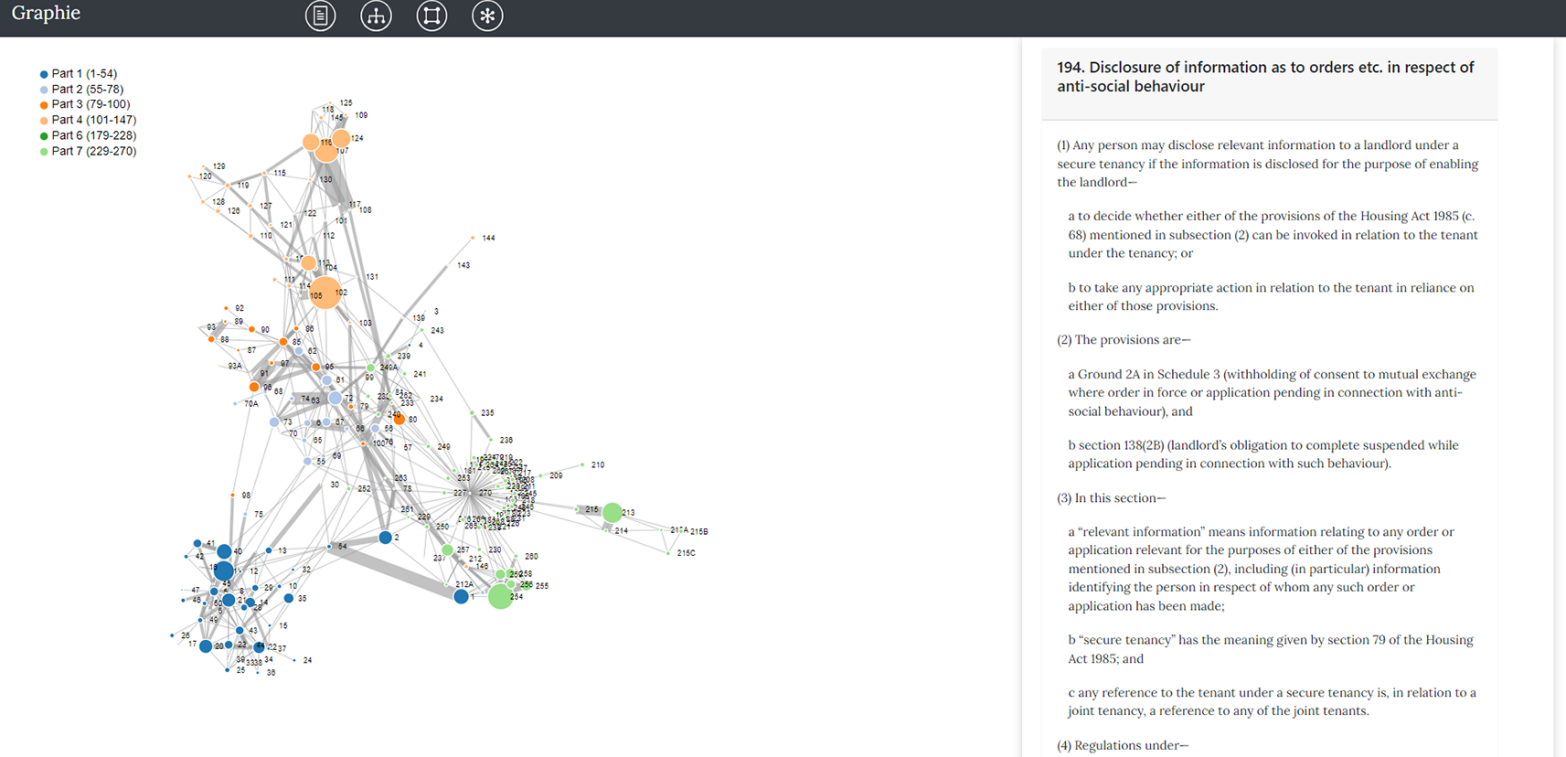
An inbound weighted representation of the Housing Act 2004 in Graphie. On the left, the web of provisions joined by a link whenever two of them can be reached in one-hop from one another. Nodes are color-coded (and the shape of the node marker can be changed too) to reflect – in this particular case – which Part of the Act each node belongs to. Hovering with the mouse over each node retrieves the textual content embedded in the node (on the right). Moving from each provision to its ‘neighbors’ no longer requires refreshing or clicking on hyperlinks, but simply wandering around with the mouse over the nodes of interest.

Thus, there is a need for improved tools and visualizations to help both occasional and professional users manage potentially demanding explorations into legal documents. This is exactly the aim of Graphie [ 7], ^26^ which provides a different and more attractive palette of network visualization tools (see an example in Figure 2) that may prove useful for law researchers and practitioners, as well as for the general public.

Before describing the main technical features and capabilities of Graphie, we put our enterprise in the wider context of Legal Map systems and similar initiatives, highlighting overlaps and differences.

**Related work**

The philosophy and technical construction behind Graphie do not write on a blank slate. The concept of a *Legal Map* system and its theoretical framework were already introduced in Ref. 4. Legal Maps are multi-layered systems offering an end-user experience similar to the user interfaces provided by geographic navigation systems, such as Google Maps [ 8]. From a mathematical perspective, a Legal Map is a directed multi-graph (network) that represents all the structural elements of a legal source. Reference 5 provides an illustration of a Legal Map network, which quantifies the legal sources of the European Union. Legal Map visualizations could unlock dependencies between legal entities that may be difficult to extract otherwise, from identifying the most “important” nodes in a network, to clustering nodes according to a given notion of similarity.

The use of tools from complexity science and network theory to analyze and represent legal texts has a relatively short but already fruitful history: arguably, the newly minted “Physics of the Law” field ^6^ will play the same role to Law that Econophysics has to Economics, ^7^ and Mathematical Biology to Biology (e.g. Ref. 8) in terms of cross-fertilization of ideas between distinct domains. An extensive graph-theoretic approach to the EU legislation network is given in Ref. 5. In Ref. 9 the tree-hierarchical network of the U.S. Code is examined by considering several scoring and ranking metrics. Reference 10 builds a hierarchical model of information (distributed on the nodes of a tree) and defines a notion of “structural complexity” on the basis of the average time a random reader takes to retrieve some piece of information planted in the leaves.

The authors of Ref. 11, prior to applying a network-driven analysis against the statutes and regulations in the United States and Germany, perform several pre-processing steps on their underlying raw data. It has been noted in Ref. 12 and in Ref. 13 that legal documents often lack sufficient metadata and are presented as plain text, creating difficulties for their application in legal informatics. We undertake a similar data preparation exercise against our raw data in Section 2.

Network-based representations of “information” do exist in other contexts, for instance academic papers. Scholarly archiving systems (Semantic Scholar [ 9], PubMed [ 10], Arxiv [ 11]) use state-of-the-art AI and API engineering that make the processing of scientific documents easier. This enables the development of applications such as Connected Papers [ 12]. Connected Papers is a network-driven citation analysis tool for enabling end users to explore relevant academic papers. The tool facilitates collecting and analyzing academic references from the chosen archiving system. Network visualizations in Connected Papers are developed using D3.js [ 13], a well-established JavaScript library for producing bespoke and interactive visualizations. With Graphie, we aim to develop a similar tool, using the same front end technology, but tailored to a different – and arguably less malleable – type of raw data. ^14^ Indeed, the XML versions of the legal texts provided by Ref. 2’s API (footnote 4) have a complex sub-structure, which is markedly different from the short-text nature (say, titles and abstract) typical of academic papers handled by citation APIs. Thus, Graphie faces the extra challenge of having to parse and build a unified model of long and intricate legal documents starting from their (XML) raw representation.

Coming back to the legal platforms field, LAWSampo ^13^ is an example of a modern legal semantic web portal in the context of the Finnish Legislation. LAWSampo is built according to the FAIR ^15^ principles of the Sampo Model ^16^ and the SAMPO-UI, ^17^ a full-stack Javascript framework. LAWSampo’s architecture clearly separates the user interface (SAMPO-UI) from the underlying Linked Data service via a SPARQL API [ 14]. Software developers or legal analysts could use SPARQL endpoints and query LAWSampo’s data service for their own Python or R applications (say network visualizations), using Jupyter notebooks. SAMPO-UI supports network visualizations by including a Cytoscape.js [ 15] based component, already demonstrated in a few portal instances of the Sampo model, existing in LetterSampo [ 16] and in AcademySampo [ 17]. In Graphie, we aim to develop the “Visualization” feature, also mentioned in Ref. 18, where sections or provisions of one Act are represented as nodes, and their “connections” along the information hierarchy as edges. Each node is endowed with its own primary XML schema reference (described below).

To facilitate the web development of LAWSampo, the authors in Ref. 13 completed a specific data exercise by initially transforming legal documents hosted on Finlex’s Data Bank server Finlex into a Linked Open Data (LOD) repository, named Semantic Finlex. ^19^ Consequently, Semantic Finlex’s data were converted into a data format compatible with LAWSampo’s semantic portal. While we do not use the concept of LOD in our work, we took a similar data pre-processing exercise in Graphie. Prior to any visualization, in Section 2 we illustrate how XML raw data from Ref. 2 are parsed and then checked against data quality indicators. The Graphie Data Model is not strictly following the FAIR principles, as it is not published as an open ontology.

## 2. Methods: The Cross-Act pipeline, Sofia

**Figure 3.**
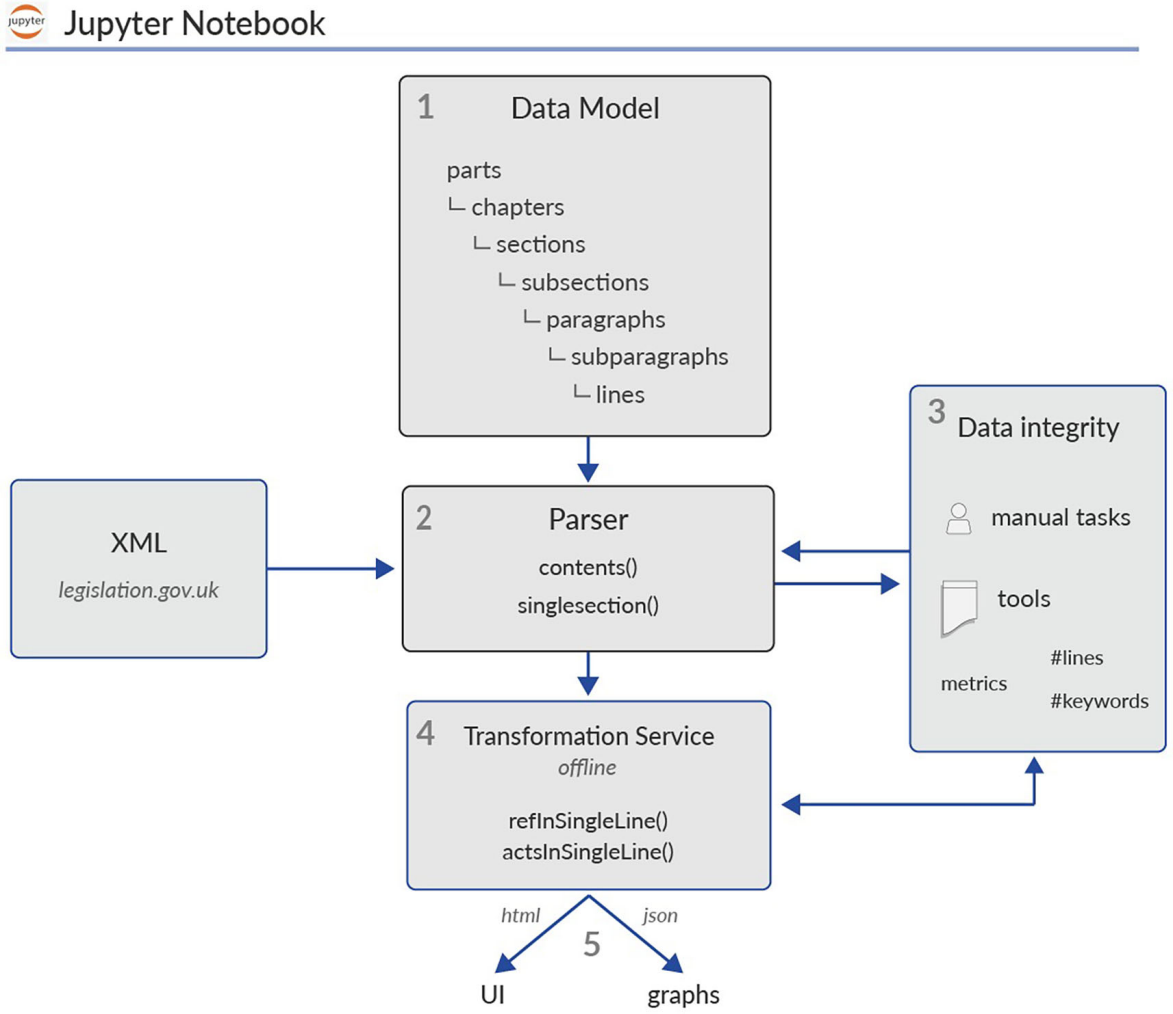
Sofia is an offline, Python-built pipeline developed as a Jupyter notebook. To implement this pipeline, we apply a multi-phase procedure that includes the following: data modelling, parser, data integrity, transformation service and visualizations. Data integrity routines are performed and hosted outside the mentioned Jupyter page.

Graphie’s aim is to tame the complexity of long and intricate legal texts by departing from the traditional “text + hyperlinks” philosophy adopted by most digital archives, in favor of a more holistic, network-based representation of the underlying information content. We achieve this goal by defining the following multi-phase pipeline (see Figure 3):

1. We codify and represent one Act’s hierarchical structure and its textual content using a programming language (in our case, Python). In Section 2.1 ( *Data modelling*), a Python object is accordingly declared, henceforth named: Graphie Object (or Graphie’s Data model).
2. We parse the raw XML document of one specific Act using the XML parser defined in Section 2.2 ( *Parser*). Ingested data feed an instance of the Graphie Object, defined in phase 1.
3. We undertake several data integrity procedures against the parsed textual elements of the Graphie object from phase 2 to ensure their quality and completeness. ( *Data integrity*)
4. We convert, using Python, the obtained instance of the Graphie Object from phase 3 into specific JSON files or HTML components. ( *Transformation service*)
5. We use the JSON files and the HTML components from phase 4 for finally feeding the underlying network libraries and certain HTML parts of our platform. ( *Visualizations*)

We designed this pipeline with data analysts in mind. Thus, Sofia ^25^ is implemented as a Jupyter notebook which offers a streamlined experience for capturing and processing UK Legislation XML documents. As we discuss in Section 2.3, data checking tools and activities are hosted outside the aforementioned Jupyter notebook and based on the outcome, the XML parser component of phase 2 is expected to be adjusted accordingly. For this reason, Sofia is an example of an offline pipeline in which we carry out data integrity checks on outputs of phase 2 and phase 4. In the following subsections we describe in detail the different phases of our pipeline, before discussing future work in Section 4.

### 2.1 Data modelling

Prior to any graph visualization, data modelling of the original data is required. In our case, we need to map elements of the UK Legislation XML files into a target data model, which is closer to our final network representation. This requires identifying: which elements from the original XML dataset should act as nodes, under which relation two nodes should be linked to one another, and whether we display any other numerical or descriptive information over our graph’s edges and nodes by applying related visual effects. The following paragraphs outline entities, relationships and properties of the raw data, which we codify as Graphie’s data model in Python.

**Primary data**

**Figure 4.**
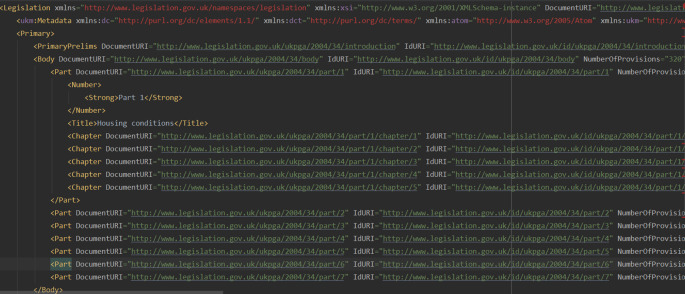
The XML tree representation of the Housing Act 2004 (see footnote 19) contains 7 tags named Parts (corresponding to the 7 parts of the same Act), and each tag Part embeds other tags, to name a few: Chapters, Pblock, Title, P1group. Subsequent P1group tags indicate the start of a section (see Figure 5).

**Figure 5.**
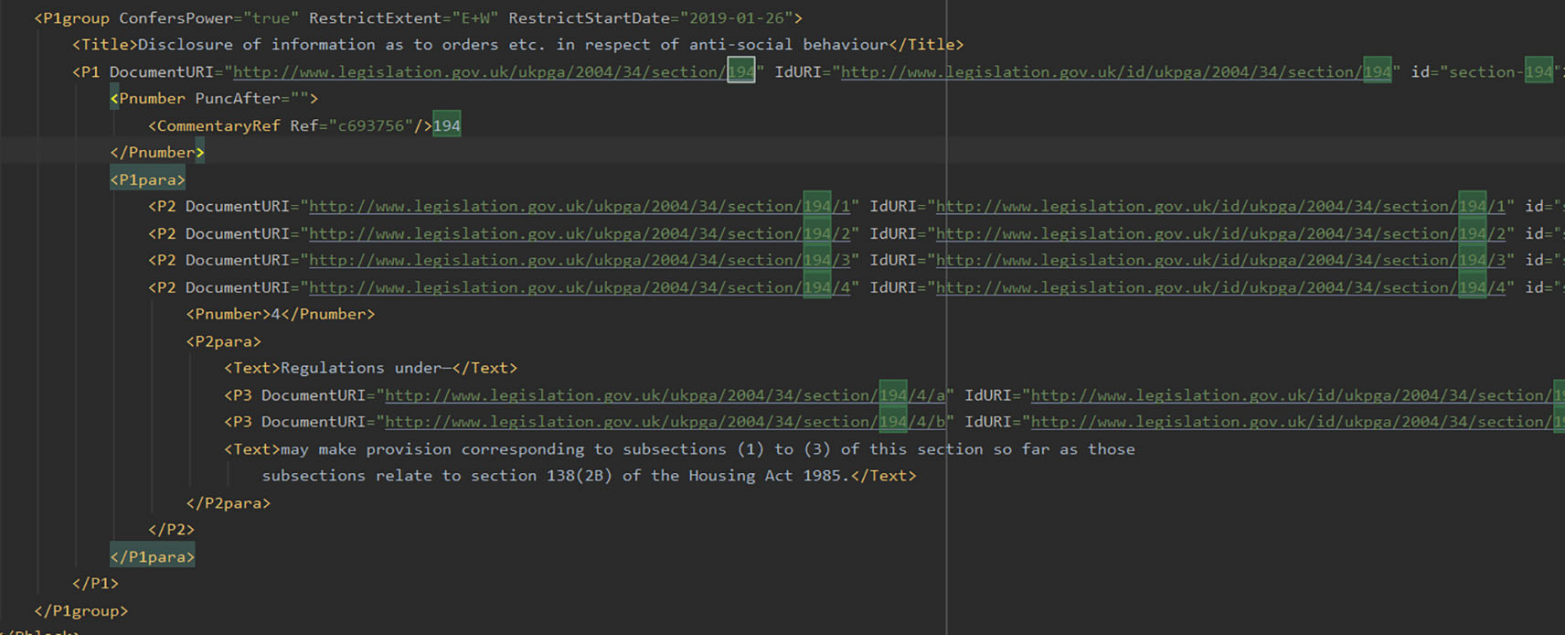
Section 194 of the Housing Act 2004, within the full data XML file (footnote 19). The content of this section is formed in a sequence (section-specific) of embedded tags: P1group, P1, P1para, P2, P2para, P3, Text. Other sections, say section 193, due to their textual complexity, might have a different tag ordering, involving BlockAmendment tags.

Each legislation page (either a whole item, or a part, or a section) on Ref. 2, is also offered as an XML file, which we refer to as the XML URL of that page. For instance, the full data of the Housing Act 2004 [ 18] is also available as an XML file [ 19] which can be referenced at footnote. Legislation XML files use the syntax of the Crown Legislation Markup Language (CLML) syntax and the associated schema. The XML files of the UK Legislation API are structured in two essential layers, the metadata layer and the content layer. Quoting from Ref. 20, “the CLML model incorporates versioning and facilitates the notion of expressing changes over time which helps us to understand the underlying metadata semantics behind the surface content”. The metadata layer is formed by elements such as: title, publication, type, format and a rigid set of persistent URIs. The content layer contains a mix of *hierarchical* ( Parts, Chapters) (see Figure 4) and *textual* tags ( Pblock, P1group) (see Figure 5). In this paper, we only focus on the content layer. The complexity of the **CLML** schema is investigated in Ref. 21 and demonstrated in Figure 3 of Ref. 20.

In Graphie we wish to visualize Acts as networks, where each section is connected with other sections if and only if there is clear textual reference between them. Consider the following last line from section 194 “… so of this section so far as those subsections relate to section 138(2B) of the Housing Act 1985…”, in Figure 12. In the same figure, the mentioned dependence is pictured as a link connecting the node representing the section 194 and the node representing the Housing Act 1985.

In the following section we define a minimal data model, sufficient for capturing both the hierarchical and textual elements of one Act’s XML structure.

**Graphie data model (or Graphie object)**

**Figure 6.**
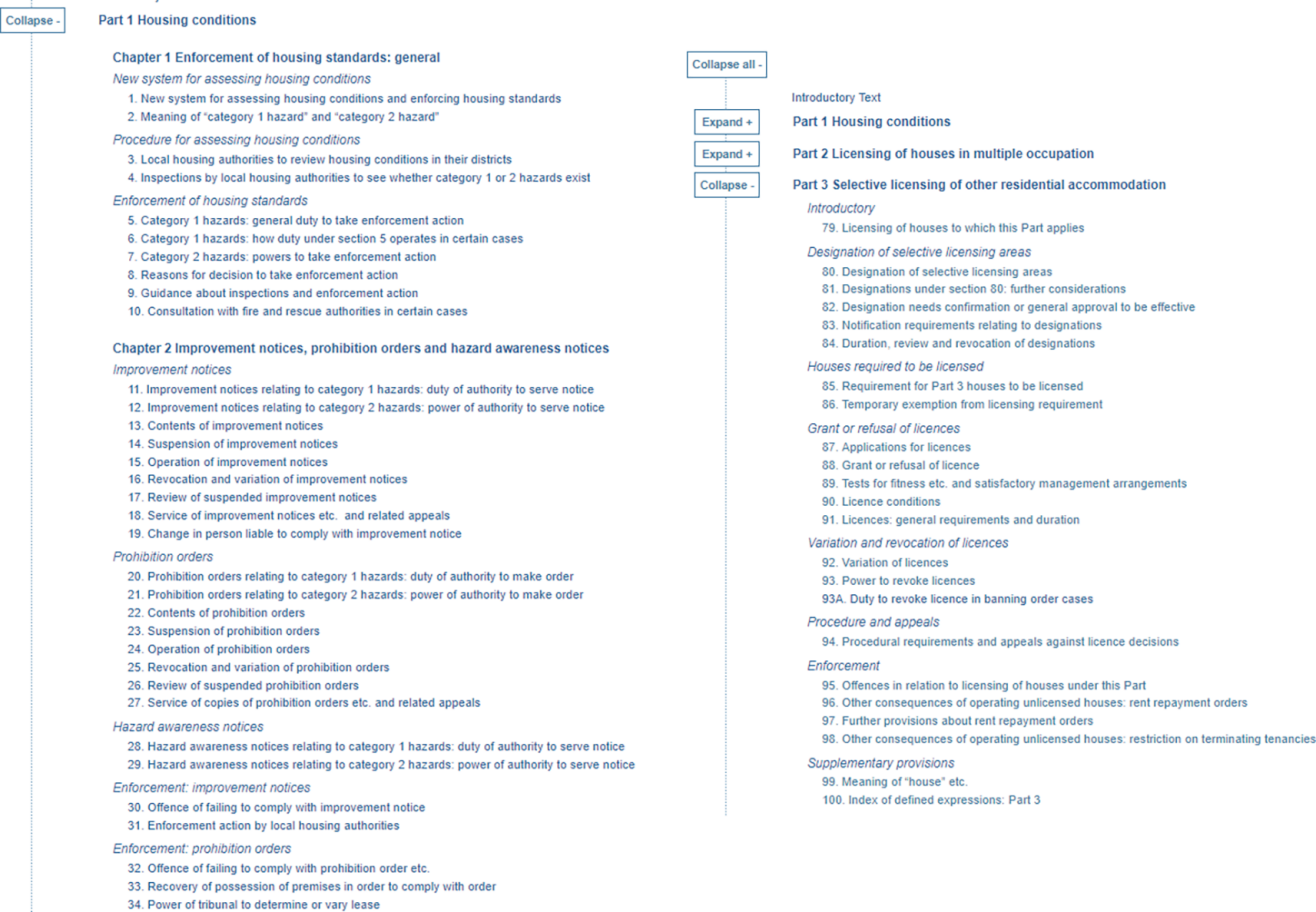
The Housing Act 2004’s table of contents as provided by Ref. 2. The Act is organized in 7 Parts, following the Legislation Structure rules of Ref. 22. Sections in Part 1, shown on the left side, are located below cross-headings. Chapters in Part 1 include a series of cross-headings. Part 3 contains only cross-headings, but no Chapters.

The UK primary legislation is structured in Acts and each Act includes different levels of division (such as Parts, Chapters, cross-headings) in a certain order, a hierarchy outlined in Ref. 22. The Housing Act 2004 shown in Figure 6 includes seven numbered parts. Each Part contains numbered Chapters and cross-headings. Chapters are above cross-headings. Cross-headings are not numbered, are displayed in italics and highlight a group of sections beneath it. Part 1, as an example, contains Chapters and Chapters include cross-headings and each cross-heading contains sections. Part 3 instead, contains cross-headings but not Chapters.

Within the structural hierarchy defined in Ref. 22, sections are the lowest level of a piece of legislation. On the textual level, they contain sub-items organized in paragraphs that might include further numbered items. This textual structure is well captured by the related definitions from section 16 in Ref. 23. Section 194 ( Figure 1) consists of: a number (194), a heading (“Disclosure of information as to orders…”) and its content. Section 194’s content is divided into four numbered subsections – (1), (2), etc. Each subsection starts with an introductory line and then contains two or more paragraphs. Subsection (3) contains three letter ordered paragraphs. Subparagraphs are usually numbered “(i), (ii)…” and are nested within Paragraphs. For instance, consider section 97 [ 20] and its subsection (6) that contains two subparagraphs (i), (ii) within paragraph (b). Conjunctive words, such as “and” or “or”, might be used for transitioning between paragraphs and subparagraphs. The canonical fine substructure of a section is suggested in Ref. 23 such a refined definition is however missing from Ref. 22. In contrast, the UK Legislation XML documents, with few exceptions, are structured according to the section logic of Ref. 23.

In Figure 3, the legal hierarchy of Ref. 22 is included within the Data Model component and is represented by the following objects: Parts, Chapters and cross-headings, whereas the textual structure mentioned in Ref. 23 is embedded within the object Sections and includes a four-level object hierarchy: SubSections, Paragraphs, SubParagraphs and Lines. Lines are the lowest element of this textual hierarchy. Thus, instances of the Graphie model meet the three specific graph model criteria ( *hierarchy*, *sequence*, *reference*) outlined in Ref. 11 (in section 2.1):

1. Elements included in one instance of the Graphie model are hierarchically structured.
2. One element’s text value is always embedded within an object of a higher-level position. These objects should also be sequentially ordered.
3. One element’s text might contain words for expressing cross-references in other sections of the same Act ( *inbound references*) or for pointing to sections from other Acts ( *outbound references*).

We implement the details of the third criterion in Section 2.4. Methods refInSingleLine() and actsInSingleLine() are used for capturing a section’s cross-references and citations, respectively.

### 2.2 Parser

In this section, we will walk through the main methods of the Python XML parser given in Figure 3. The parser is developed using the beautifulsoup [ 21] library, a well known Python tool, which allows you to try out different web scrapping strategies.

**Table 1.** Graphie’s data model main objects. Table numbers refer to the obtained instances for each object after parsing Housing Act’s 2004 full XML content. The reported numbers are heuristically calculated and do not include textual information about sections declaring legislation amendments.

Object	Instances	Notes
Parts	7	Housing Act 2004 is organized in 7 parts.
Chapters	14	Parts may contain Chapters.
cross-headings	92	Within a **Part** or under a **Chapter**, a sequence of Cross-Headings.
Sections	281	Sections are numerically enumerated, where few section names might include capital letters A,B and C. Say, 215A, 215B and 215C.
SubSections	1410	Sections are usually divided into numbered subsections ((1), (2), etc).
Paragraphs	1783	Subsections may contain enumerated paragraphs ((a), (b), ….)
SubParagraphs	242	Paragraphs may include numbered ‘(i), (ii), …’ SubParagraphs.
Lines	32	SubParagraphs might be subdivided into single lines.

Consider the “table of contents” page ( Figure 6) of the Housing Act 2004 [ 22]. As expected, both the underlying XML document and the displayed HTML page are structured in Parts, Chapters and cross-headings. Each section’s titles are hyperlinked, and each hyperlink points to one section’s whole web document. For instance, in Figure 6, the title of section 3, “Local housing authorities to review housing conditions in their districts”, is located below the cross-heading “Procedure for assessing housing conditions” and points to the individual web page for section 3 [ 23].

The two fundamental components of our parser are the contents() method and the singleSection() method, both of which are defined below. Each method can be executed in isolation. The role of contents() is to map the legal hierarchy structure included in an Act’s “table of contents” XML page into the following items of the Graphie Object: Parts, Chapters, cross-headings (mentioned in Section 2.1). Given a section’s XML URL and using the singleSection() method instead, we can capture and map the textual content of a section to the following elements of the Graphie Object: Sections, Paragraphs, SubParagraphs and Lines. Both methods are not Act-specific and will be discussed elsewhere in this article as well, such as in the context of Data Integrity (Section 2.3) and in the Transformation Service (Section 2.4) sections.

**contents()**

**Figure 7.**
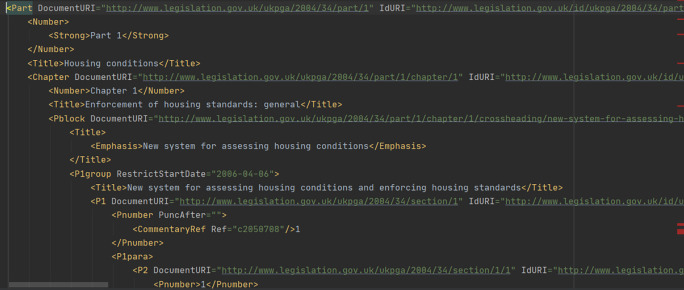
The Housing Act 2004 – the XML representation of the full data XML file as provided by Ref. 2. The file contains information about same Act’s components: Parts, Chapters, cross-headings and sections.

The “Table of Contents” page is where legislation readers could get an idea of how an Act is organized according to the legal hierarchy outlined in Ref. 22. The “Table of Contents” web page for the Housing Act 2004 (footnote 22) is divided into Parts, Chapters or cross-headings and sections. All sections have headings alongside their numbers, and are grouped within a Chapter or a cross-heading. The above structure is well identified in Figure 7, corresponding to an Act’s full data XML file: A Part is the root element for a group of Chapters. It has has a number and a title. Chapter tags have numbers and titles, too. A Chapter often includes cross-headings. A Pblock start tag indicates a cross-heading, and each cross-heading has a name. A cross-heading usually contains two or more sections. Sections are defined as P1group tags and always contain other elements such as Title, PNumber and DocumentURL elements.

**Figure 8.**
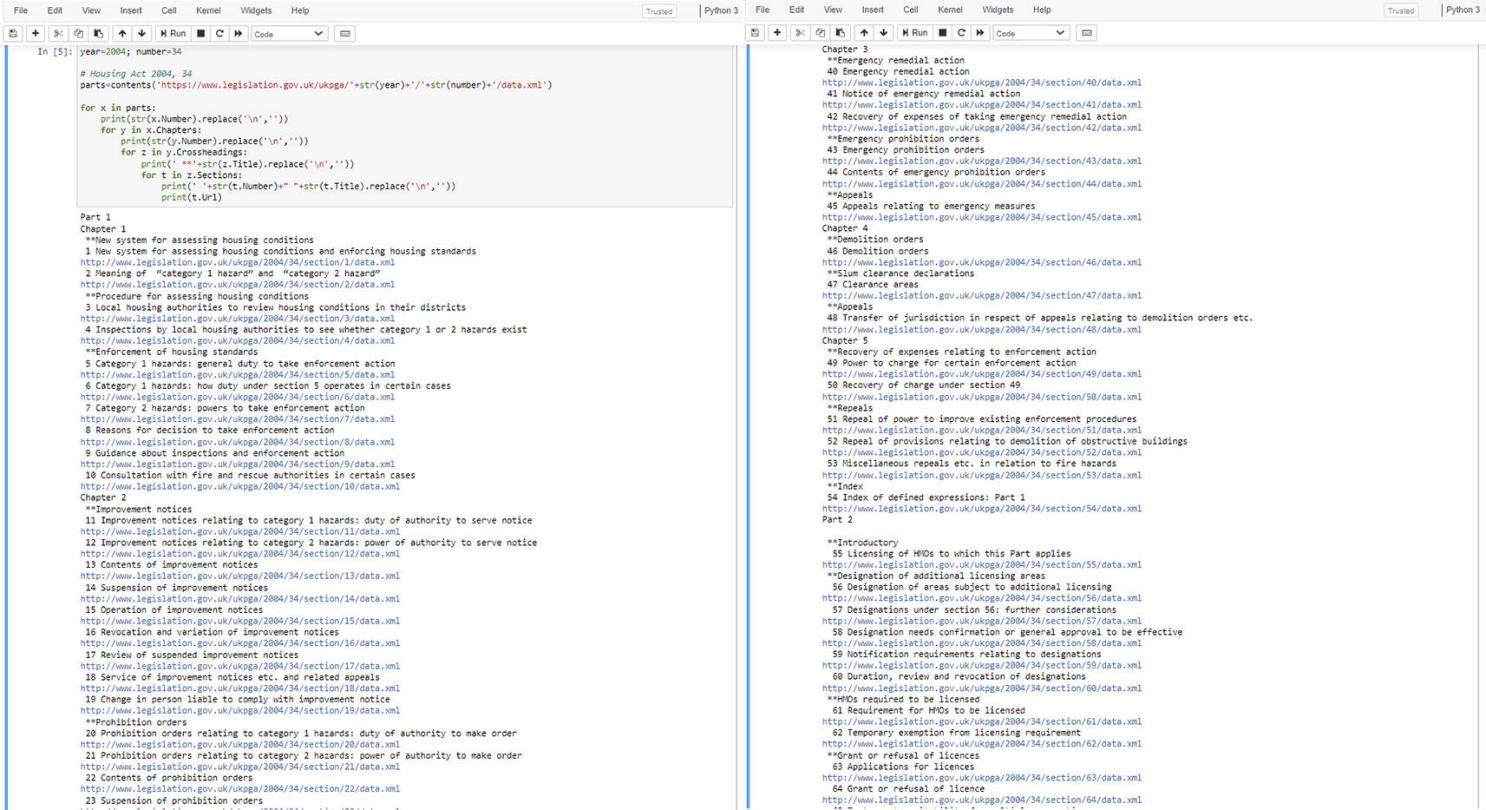
The Housing Act 2004 – Table of Contents. contents() is a function with one argument url. When contents() is called, we pass along the URL corresponding to one Act’s full data XML file. This URL is used inside the function for returning an instance of the Python object Parts that contains nested occurrences of the following objects: Chapters, cross-headings and Sections.

For indexing an Act’s legal structure, we use the method contents(). The parsed table of contents of the Housing Act 2004 is shown in Figure 8. The method contents() is clearly able to handle the structural variety between Part 1 and Part 2 (no chapters, only cross-headings).

At this stage, one can easily declare a new array variable, say urls, for storing all the DocumentURL elements returned by contents() (see Figure 8). In the next section we discuss how we could fully XML-scrap an Act’s content by running the next defined method singleSection() for each DocumentURL in urls.

**singleSection()**

**Figure 9.**
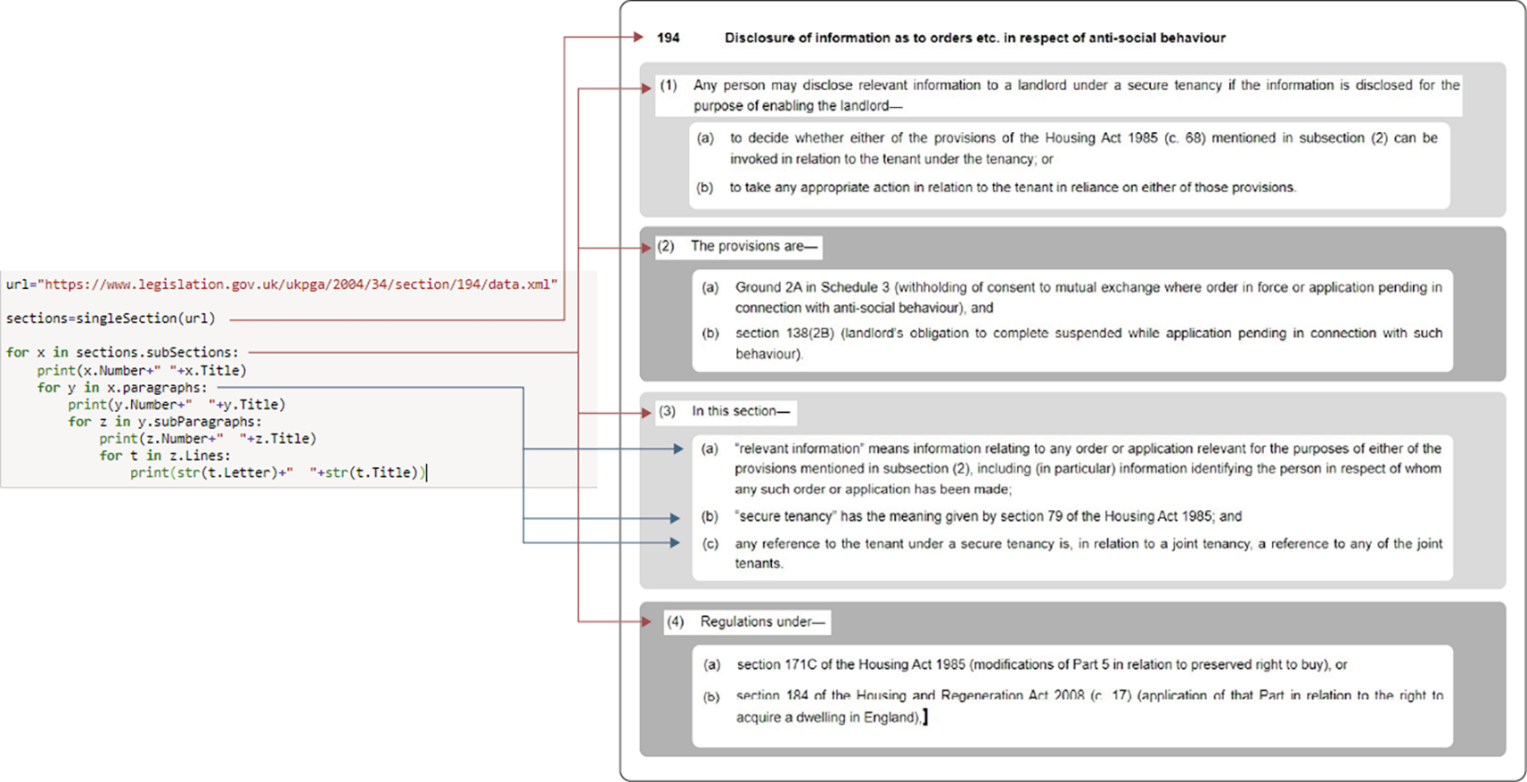
The textual on memory representation of section 194 after parsing its content using the method singleSection(). The obtained Sections instance contains embedded objects ( SubSections, Paragraphs, SubParagraphs and Lines) which are hierarchical and sequentially ordered: section 194 contains 4 subsections. Each subsection includes ordered paragraphs.

The aim of the singleSection() method is to map, applying the text-structural rules of Ref. 23, a section’s (or subsection’s) content into an instance of the Python object Section defined in Section 2.1. In Figure 9, the object Section captures section’s 194 content in four instances of the object SubSections, reflecting the four numbered items in Figure 1. Each instance of SubSections consists of an array of Paragraphs objects, corresponding to the letter numbered lines. In our example, the SubSections object of the third item is composed of three Paragraphs, each paragraph corresponding to one item of the ordered list (a), (b) and (c).

Following legislation amendments, one section’s subsection can change another Act’s sections content. ^23^ Consider that due to the use of the word “insert” in subsection (4) of section 185 [ 24] of the Housing Act 2004, section 155 of the House Acting Act 1985 is modified by inserting two new sections, sections 155A and section 155B. The word “substitute” (see subsection (2) in section 185) is also used for such amendments. In our data model (see Figure 3) sections embed subsections and subsections incorporate paragraphs. Since amendments modify sections, they may be seen as superior objects to sections; on the other hand, their text is usually located within subsections. Our data model does not allow for sections to be subordinate to subsections, and is therefore unable to fully express amendments (see Section 2.3).

singleSection() is heuristic in nature and depends on the underlying structural variety of the Legislation XML schema. For achieving high levels of accuracy, singleSection()’s output should be manually or programmatically checked, as we explain in the next subsection.

### 2.3 Data integrity

The aim of the XML parser developed in Section 2.2 is at least twofold: firstly, we wish to speed up the data collection process and secondly, we plan to use the same parser for collecting data from other Acts. Data integrity is captured as a dedicated component in the flowchart of Figure 3, a component that requires the attention of data analysts well trained in various tools. In our scenario, prior to any visualization, we are looking to improve our XML parser’s output by incorporating related data quality observations.

**Figure 10.**
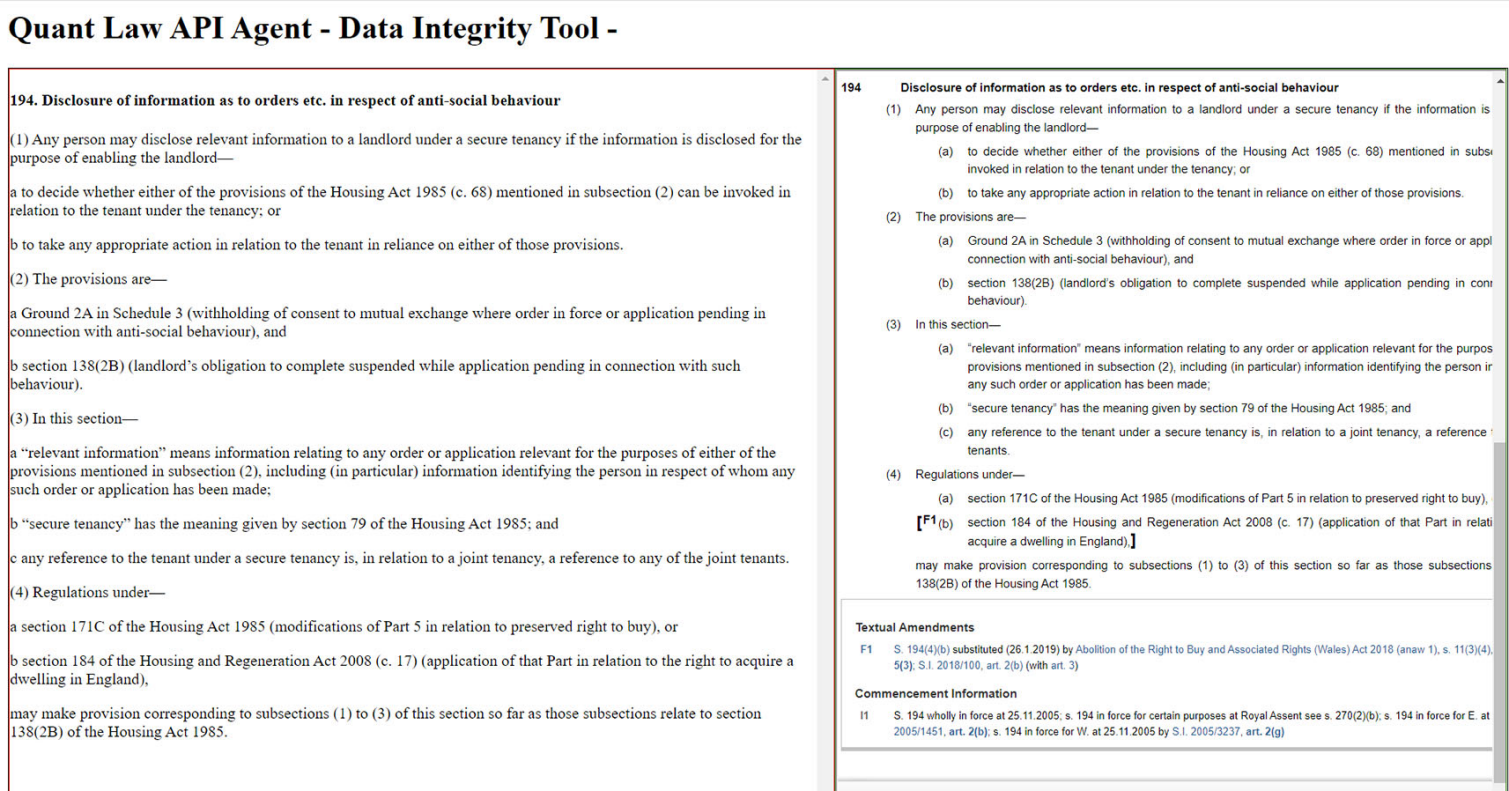
A screenshot from the bespoke data quality tool, developed for replacing quality checks based on visual comparison on two different monitors. On the left, section 194’s parsed content. On the right, the same section’s content in Ref. 2.

Consider a data quality engineer parsing the XML file of section 194 of the Housing Act 2004. Following the steps of our pipeline, they would need to inspect singlesection()’s output (displayed on a Jupyter window) against the original content of the same section on legislation.gov.uk (an HTML web page). Practically, this would require a visual comparison between two different pages on a browser. For facilitating speedy comparisons between parsed and original data, we developed a bespoke data integrity web tool. In Figure 10, the left frame displays one section’s parsed content. The same section’s original content (shown in Ref. 2) is located on the right frame. This blended visualization between original and parsed data makes their comparison easier and faster. Otherwise, we would have to keep two full screens open for comparing parsed and original data. Other key variables that we could use for evaluating our parser’s performance are the total number of subsections, paragraphs, sub paragraphs and single lines included within one section.

Regular expressions are useful for data engineering routines, also used in the preprocessing steps of Ref. 14. In Table 2 we report the frequency of a few important expressions after running Regex Search [ 25] on the Housing Act 2004’s one-page view (see footnote 18). Consider the regular expression “ *of the [aA-zZ]+ Act*”. This regex is used for finding all Act names mentioned within the Housing Act 2004 and reports 156 such instances (outbound references). In Table 5, prior to network visualization in Figure 12, we match these instances with the output obtained by our parser’s method: actsInSingleLine() (see Section 2.4). “ section [1-9]* or [1-9]” identifies those lines (say, subsection 1 in section 13 [ 26]) which include a section number (inbound reference), an “or” and are followed by a numerical digit from 1 to 9, pointing to other sections. For the network shown in Figure 2, this is a useful pattern as it helps us to pin down and review edges corresponding to sections related by an “or” conjunction.

**Table 2.** Results of single line regular expression searches, executed using Regex Search (see footnote 25) on one page’s view of the Housing Act 2004. A data engineer can use these patterns as alternative metrics for reviewing our parser’s performance about cross-referencing and citations collection.

Regex	Occurrences	examples
section [1-9]*	500	1(2b)
section [1-9]* or [1-9]	20	13(1)
section [1-9]* or section [1-9]	3	102(10)
section [1-9]* to	21	1(5)
sections [1-9]* and	4	105(11)
*of the [aA-zZ]+ Act*	156	1(5)

### 2.4 Transformation service

**Figure 11.**
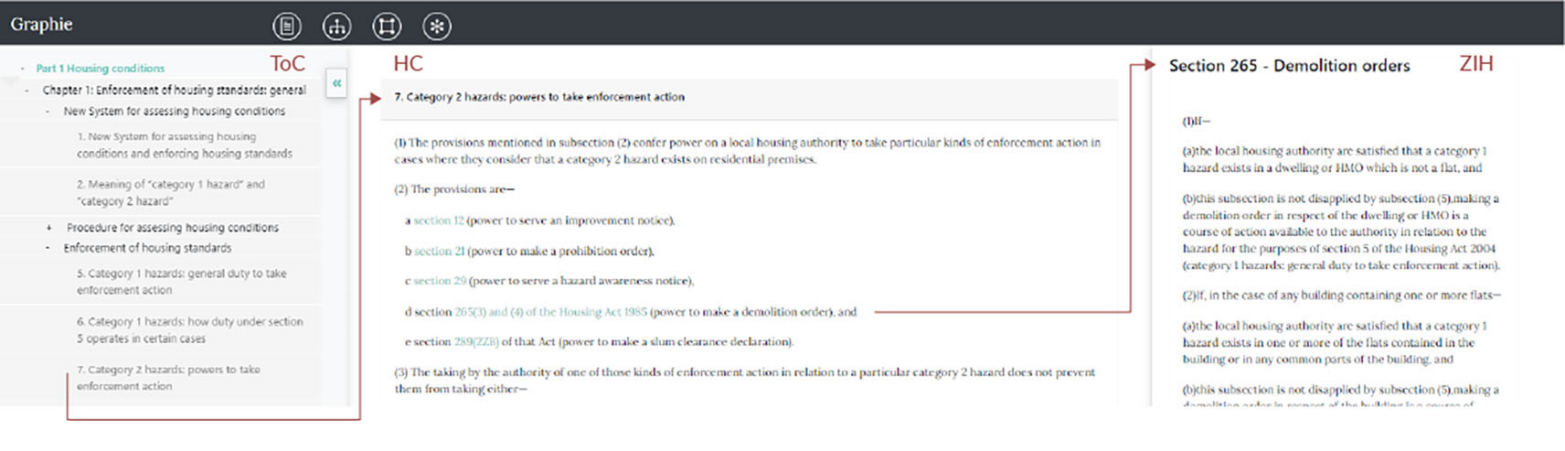
An instance of Graphie’s landing page. The user visualizes section 7 after clicking on the lowest link within the table of contents of Part 1, located on the left panel (ToC). Subsection 2(d) (within the middle panel, (HC) contains a hyperlinked outbound reference to section 265 of the Housing Act 1985. This hyperlink enables users to display section’s 265 content on the right panel (ZIH). All of these panels - ToC, HC, and ZIH - are generated by the related methods shown in Table 3.

At this point of our data journey, we should expect that original XML legislation files are now accurately represented on memory as an instance of the Graphie Python object, defined in Section 2.1. In Figure 11, we show on the left the table of contents generated using a specific instance of the Graphie object, mirroring the Housing Act’s 2004 table of contents (footnote 22). The JSON file feeding the graph visualization of Figure 2 is also generated based on the aforementioned Graphie object.

The transformation service is a library of Python methods for serializing the memory representation of an Act into customised JSON or HTML outputs. Checking data integrity over this marshalling process is a crucial part of a data engineer’s job. A data engineer will first automatically translate the on memory data into a specified format, and then they will consequently review the data validity of the obtained output. The relation between data integrity checks and the transformation service is also reflected in Figure 3.

refInSingleLine() and actsInSingleLine() are two fundamental functions of the transformation component of our pipeline. Each function identifies string cross-references ( refInSingleLine()) or citations ( actsInSingleLine()) to other Acts from a text string.

Let us take as an example the single line of subsection 2(a) in section 7 ( Figure 11): “section 12 (power to serve an improvement notice)”. Use this line as an input parameter to refInSingleLine(), and section 12 is returned. Thus, section 7 and section 12 form an *inbound reference* that we also express as a dedicated link between node 7 (section 7) and node 12 (section 12) in Figure 2. The repeated application of method refInSingleLine() for each line of section 7 returns the following array: [“section 12”:1, “section 21”:1, “section 29”:1]. The array indicates three single inbound references, from section 2 to sections 12, 21 and 29. Such calculated metadata values are also included in inbound.json [ 27], the underlying file of the inbound complexity network of Figure 2. Similarly, we have also found that the most frequently referenced section in Housing Act 2004, is section 102 ( Table 4).

**Table 3.** Methods used to extract elements of the full data XML files, and where the results are visualized in this manuscript. In Figure 2, on the left, the table of contents is generated by the method divNav(). The method htmlSingleSection() instead prints one single section’s content as a customized html paragraph of our platform. We use the methods refInSingleLine() and actsInSingleLine() for picking out which nodes (sections) should be linked together with an edge in Figures 2 and 12.

Element	Figures	Method
Table of contents ( **ToC)**	Fig. 11	divNav()
Single Section ( **HC)**	Fig. 2, Fig. 11	htmlSingleSection()
Inbound Complexity Graph, JSON file	Fig. 2	refInSingleLine()
Outbound Complexity Graph, JSON file	Fig. 12	actsInSingleLine()

**Table 4.** Frequently referenced sections of the Housing Act 2004. A table showing the top 5 (out of 281) most referenced sections of the Housing Act 2004, their reported frequency after repeatedly applying refInSingleLine() to each single line of the same Act and the number of unique references after manually inspecting the Housing Act 2004’s full web content (see Section 2.3).

Section	refInSingleLine()	Data Integrity
102	19	25
254	15	15
107	14	20
11	13	24
213	12	20

**Figure 12.**
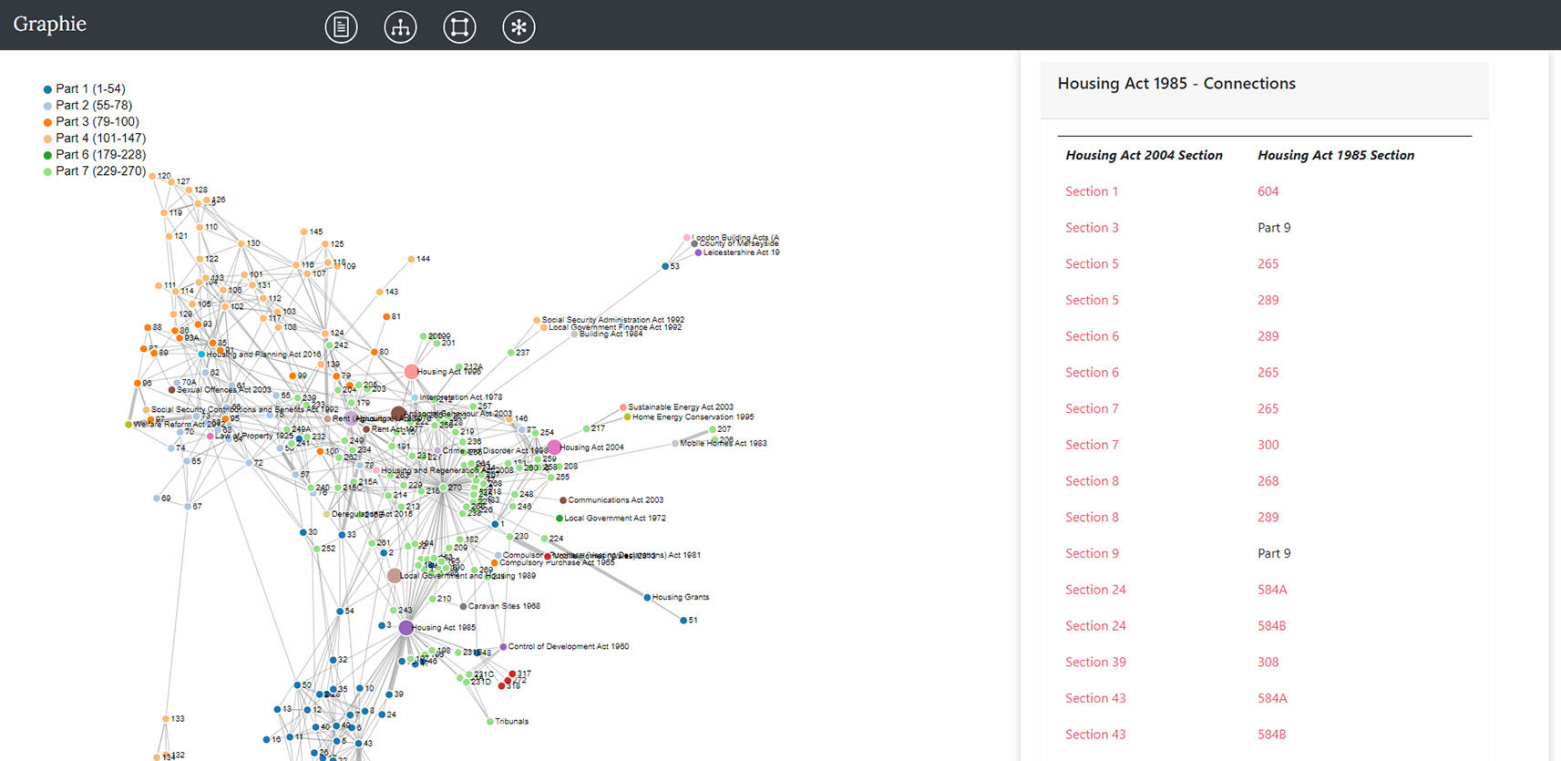
Our full legal network representation of the Housing Act 2004 in which nodes are connected with others if they share inbound or outbound references. In this instance, the user could click on “the Housing Act 1985” node and fetch on the right (ZIH panel), all the outbound references connecting the Housing Act 2004 with the aforementioned Act. Section numbers are hyperlinked and users can continue exploring sections from both Acts.

Subsection 4(b)’s text in section 194 ( Figure 1) is now passed as a parameter to actsInSingleLine() and “the Housing and Regeneration Act 2008” title is returned. Thus, section 194 contains an outbound reference to the Act just mentioned. Call actsInSingleLine() on every line of section 194 and you will obtain the following array: [“Housing Act 1985”:4, “Housing and Regeneration Act 2008”:1], highlighting all the outbound references within section 194. The value 4 next to the key the Housing Act 1985 is a calculated metadata value that counts how many times the Housing Act 1985 is textually mentioned within section 194. The same value determines that edge’s weight (henceforth, thickness) in Figure 12. One edge’s thickness is also represented in outbound.json by the attribute “thick” (same attribute is used in inbound.json). Thus, the outbound.json file populates information about nodes and links shown in Figure 12, a network that highlights the outbound connections between sections in Housing Act 2004 with sections from other Acts.

UserMark [ 28] is a similar and well-matured mark-up tool, that allows users to create hypertext links from any text to AustLII’s [ 29] legislation and High Court corpus.

## 3. Use cases: Visualizations

In this section, we illustrate how Graphie could be used in practice. Graphie offers a few main functionalities: a One Page View ( *OPV*) navigation, and two network visualizations. These representations could be a powerful tool for portraying the legal understanding of an ideal law practitioner that knows all the interconnections and shortcuts among different sections and Acts of the UK’s Statute Book. While similar visualizations could be obtained by using native Python or R network packages, like, “networkx” [ 30], network analyses are usually published using browser friendly tools, such as D3.js, Cytoscape [ 31], KeyLines [ 32]. For example in Ref. 24, initial network analyses were completed in “networkx” whereas their output was produced in Cytoscape. Other examples are networks in Sampo-UI, which exploit Cytoscape.js [ 33] mechanisms and features: zooming, panning, node sizing, node coloring, and directed edges. In Graphie we implemented, using native D3 libraries [ 34, 35] a novel network navigation framework that allows users to click on a node and display that node’s underlying textual content. Such advanced mouse functionalities are not supported by default in Cytoscape.js and require further development.

### 3.1 One-page view

The landing page of Graphie, depicted in Figure 11 offers a *One-Page View* (OPV) of the Housing Act 2004. OPV displays a three-part canvas made up of the following panels: Table of Contents ( *ToC*), highlighted contents ( *HC*), and zoomed-in hyperlinks ( *ZIH*). On the left, users can explore the ToC of the Housing Act 2004 by expanding the related tree hierarchy. By clicking on a ToC link, the user can see the actual content of the chosen section in the next panel (HC). Let us go over an example: A user clicks on the ToC link of section 7 ( Figure 11) and that section’s content is shown within the HC area. Section 7 is connected with several other sections, and the user may now click on the link of section 265 of the Housing Act 1985 (outbound reference). This section’s content is then pulled up on the right ZIH panel. All mentioned panels (ToC, HC, ZIH) are visible at all times. Thus, OPV enables users to review different sections on one single page, without switching between different pages (as experienced in Refs. 2, 3). Also, sections are not hyperlinked in one Act’s “full text” page on legislation.gov.uk (see footnote 18). Further, the OPV feature is not offered in Westlaw. ^3^ In Graphie instead, section numbers are hyperlinked.

From a technical point view, ToC displays the table of contents of the Housing Act 2004 as a nested ordered list. The HTML content of ToC is generated by the method divNav(), whereas sections in HC and ZIH are built by the method htmlSingleSection(). All mentioned methods are part of the Transformation service, see Section 2.4. For more details on the methods used for visualization, please refer to Table 3.

### 3.2 Network view – inbound complexity

By clicking on the square navigation icon in Figure 2, a network graph representation of the Housing Act 2004 is opened. In this network, only sections of the same Act (inbound references) are connected and displayed. Each section’s raw XML data is analyzed for cross-references using the method refInSingleLine(), defined in Section 2.4. We use the former method’s output for creating the json file inbound.json that includes clear information about this network’s nodes and their connections. The same file is used for populating our network front-end library with data. The obtained network consists of 281 nodes and 517 edges. In inbound.json, each node record and each link record are associated with metadata variables, such as “nodeSize” and “thick”. Edge thickness in Figure 2, depends on “thick” values (also defined in Section 2.4). Node size instead, is set according to “nodeSize” and reflects the total count of references found within the Housing Act 2004 about that node’s section number. That is, section 194 appears only once in section 270, subsection 5(c). Thus, section 194’s node size is 1.

Node coloring works as follows: each Part is assigned with a colour, and any nodes within this Part should be assigned the same color. For instance, section 1 of Part 1 is denoted as the blue node 1. All sections (nodes) of Part 1 are coloured in blue. The user can still click on one node’s circle, and display on the right (ZIH panel) their hyperlinked content.

The network reports strong connectivity between same coloured nodes, indicating that sections of the same Part are expected to be tightly linked together. Sections of Part 5 are content-less, thus excluded from this inbound complexity network.

### 3.3 Network view – outbound complexity

Table 5 lists the 5 most commonly mentioned Acts within the Housing Act 2004’s sections. There are 39 such Acts, obtained by applying the method actsInSingleLine() to each section of the Housing Act 2004. Each of these Acts is represented as a single, coloured node in Figure 12. This new network is obtained from the original inbound network ( Figure 2) by also connecting sections of the Housing Act 2004 with the collected new (external) Acts. The resulting graph consists of 327 nodes and 671 edges and is obtained in Graphie by clicking on the star navigation icon. The user again can click on a node and display the underlying content of the chosen section.

**Table 5.** Table showing the top 4 (out of 43) most frequent external Acts connected with sections of the Housing Act 2004. For each Act, the column actsInSingleLine() displays their occurrences found by iteratively calling actsInSingleLine() (see Section 2.4) over the full content of the Housing Act 2004, whereas the next column “Data Integrity” shows the actual observed occurrences after our data integrity checks (say, regular expression searches elaborated in Section 2.3 and Table 2).

Act Name	actsInSingleLine()	Data Integrity
Housing Act 1985	48	54
Housing Act 1988	18	18
Housing and Planning Act 2016	15	16
Housing Act 1996	13	14

## 4. Conclusions

In this paper, we designed and described a cross-Act pipeline for establishing a connection between the raw data provided by the UK Legislation API and our platform’s front-end network representations of Acts and Bills included in the UK’s Statute Book. Our networks reveal interesting associations between individual provisions, and also enable users to fully explore one Act’s content by just hovering over each node with the mouse. Nodes are associated with their underlying textual, hyperlinked content, and by clicking on one node the user can display this information on a dedicated panel alongside. Visiting different sections and hopping between provisions of an Act no long requires opening new pages via hyperlinks (or via copy-pasting of the new node’s address into the browser): the user can now remain on the same page and arrive at the information sought by simply clicking or hovering on their nodes of interest.

In our efforts to mold legislation from raw data into their final network visualizations, we faced the task of processing them carefully and accurately. We tried to automate this process as much as possible. Due to the structural variety of our underlying files and the need to calculate network-specific metadata instances (not included in our original data), we had to incorporate a supervised layer of data integrity and data quality checks.

The next challenge will be to apply the developed pipeline on a larger scale, and to go beyond the Housing Act we focused on for illustrative purposes. There are several ideas and further steps to be considered. First and foremost, we wish to use and test our parsing and data integrity routines against a larger volume and broader classes of Acts. The process of identifying references between sections (inbound or outbound) and populating related hyperlinks should be further improved and automated to reduce the need for human supervision. Due to the expected larger population of nodes, we might consider adjusting or creating new visualization tools to avoid cramming effects on the screen. Such refinements usually require long learning and development curves. Our experience working with the sections of the Housing Act 2004 has however convincingly demonstrated the proof of concept of a fully operational prototype for the network-based visualization of the UK’s primary legislation – beyond the classical text-only paradigm – which promises a fresh way to conceive, analyze, and represent legal texts in a user-friendly way.

## Data Availability

All data used for this project were publicly available and downloaded from the UK’s legislation platform. ^2^ This data is available as both full XML files and in html formatted text, as discussed in more detail in the “Primary Data” paragraph, in Section 2.

## Footnotes

^1^
Reported numbers till 2016, in Ref. 1.

^2^
Available here: www.legislation.gov.uk/new

^3^

https://www.nationalarchives.gov.uk

^4^
The UK Legislation API, https://www.legislation.gov.uk/index

^5^

https://www.legislation.gov.uk/developer/uris

^6^
This means that section 194 includes multiple references to other statutes.

^7^

https://graphie.quantlaw.co.uk/

^8^

https://www.google.com/maps

^9^

https://www.semanticscholar.org/

^10^

https://pubmed.ncbi.nlm.nih.gov/

^11^

https://arxiv.org/

^12^

https://www.connectedpapers.com/

^13^

https://d3js.org

^14^

https://www.w3.org/TR/sparql11-query/

^15^

https://js.cytoscape.org/ - a Graph theory library

^16^

https://lettersampo.demo.seco.cs.aalto.fi/en/actors/faceted-search/network LetterSampo, a network of Historical Letters

^17^

https://akatemiasampo.fi/en/people/faceted-search/network AcademySampo, a network of Finnish Academic People

^18^
Available at https://www.legislation.gov.uk/ukpga/2004/34/data.xml

^19^

https://www.legislation.gov.uk/ukpga/2004/34/data.xml, which we call the full data XML file.

^20^

www.legislation.gov.uk/ukpga/2004/34/section/97

^21^

https://www.crummy.com/software/BeautifulSoup/

^22^

https://www.legislation.gov.uk/ukpga/2004/34/contents

^23^

https://www.legislation.gov.uk/ukpga/2004/34/section/3

^24^

www.legislation.gov.uk/ukpga/2004/34/section/185

^25^

https://chrome.google.com/webstore/detail/chrome-regex-search/bpelaihoicobbkgmhcbikncnpacdbknn

^26^

www.legislation.gov.uk/ukpga/2004/34/section/13

^27^

graphie.quantlaw.co.uk/inbound.json

^28^

http://www.austlii.edu.au/techlib/usermark/

^29^

http://www.austlii.edu.au/

^30^

https://networkx.github.io/

^31^

https://cytoscape.org

^32^

https://cambridge-intelligence.com/keylines/

^33^

https://manual.cytoscape.org/en/stable/Navigation_and_Layout.html

^34^

https://observablehq.com/@d3/force-directed-graph

^35^

https://bl.ocks.org/mohdsanadzakirizvi/6fc325042ce110e1afc1a7124d087130

## Software availability

Graphie is published at: graphie.quantlaw.co.uk.

The source code used in this study is available on Github, in the following repositories:

• Sofia, a Jupyter Pipeline: https://github.com/kclquantlaw/pipeline
  Archived source code at the time of publication: https://doi.org/10.5281/zenodo.7611873 ^25^
• Web Platform’s source code: https://github.com/kclquantlaw/graphie
  Archived source code at the time of publication: https://doi.org/10.5281/zenodo.7620232 ^26^

License: MIT

## References

[1] Loft P Apostolova V : Acts and Statutory Instruments: the volume of UK legislation 1950 to 2016. 2017. Reference Source

[2] National Archives: Enacted UK Legislation. 2022. Reference Source

[3] Thomson Reuters: Westlaw UK – Online Legal Research. 2022. Reference Source

[4] Ruhl JB Katz DM : Measuring, monitoring, and managing legal complexity. *Iowa L. Rev.* 2015;101:191.

[5] Koniaris M Anagnostopoulos I Vassiliou Y : Network analysis in the legal domain: a complex model for European Union legal sources. *J. Complex Netw.* 2017;6:243.

[6] Vivo P Katz DM Ruhl JB , editors. The Physics of the Law: Legal Systems Through the Prism of Complexity Science. *Front. Phys.* 2021;9. 10.3389/978-2-88976-129-6

[7] Smolyak A Havlin S : Three Decades in Econophysics – from Microscopic Modelling to Macroscopic Complexity and Back. *Entropy.* 2022;24:271. 10.3390/e24020271 35205566 PMC8870777

[8] Codling E Plank M Benhamou S : Random walks in biology. *J. R. Soc. Interface.* 2008;5:813–834. 10.1098/rsif.2008.0014 18426776 PMC2504494

[9] Katz DM Bommarito MJ : Measuring the complexity of the law: the United States code. *Artif. Intell. Law.* 2014;22:337–374. 10.1007/s10506-014-9160-8

[10] Förster Y-P Annibale A Gamberi L : Information retrieval and structural complexity of legal trees. *J. Phys.: Complexity.* 2022;3:035008. 10.1088/2632-072X/ac8e48

[11] Coupette C Beckedorf J Hartung D : Measuring law over time: A network analytical framework with an application to statutes and regulations in the United States and Germany. *Front. Phys.* 2021;9:658463. 10.3389/fphy.2021.658463

[12] Erdelez S O’Hare S : Legal informatics: Application of information technology in law. *Annu. Rev. Inf. Sci. Technol.* 1997;32:367.

[13] Hyvönen E Tamper M Ikkala E : Lawsampo portal and data service for publishing and using legislation and case law as linked open data on the semantic web. *submitted.* 2021. Reference Source

[14] Coupette C Hartung D Beckedorf J : Law Smells: Defining and Detecting Problematic Patterns in Legal Drafting. *Artif. Intell. Law.* 2022. 10.1007/s10506-022-09315-w

[15] Wilkinson M Dumontier M Aalbersberg I : The FAIR guiding principles for scientific data management and stewardship. *Sci. Data.* 2016;3:160018. 10.1038/sdata.2016.18 26978244 PMC4792175

[16] Hyvönen E : Digital Humanities on the Semantic Web: Sampo Model and Portal series. *Semant. Web.* 2022;1–16. accepted. 10.3233/SW-223034

[17] Ikkala E Hyvönen E Rantala H : Sampo-UI, A Full Stack Javascript Framework for Developing Semantic Portal User Interfaces. Semantic Web - Interoperability, Usability, Applicability. *Semant. Web.* 2022;13:69–84. 10.3233/SW-210428

[18] Robinson D Yu H Zeller W : Government data and the invisible hand. *Yale J. L. Tech.* 2009;11:159.

[19] Oksanen A Tuominen J Mäkelä E : Semantic finlex: Transforming, publishing, and using Finnish legislation and case law as linked open data on the web. Peruginelli G Faro S , editors. *Knowledge of the Law in the Big Data Age, Front. Artif. Intell. Appl.* 2019;317:212.

[20] Kumar B Mcgibbney L : A comparative study to determine a suitable representational data model for UK building regulations. *J. Inf. Tech. Construction.* 2013;18:20.

[21] King’s Digital Lab: Taming the complexity of law: Modelling and visualization of dynamically interacting legal systems. 2022. Reference Source

[22] National Archives: FAQ, Enacted UK Legislation. 2022. Reference Source

[23] Government of Western Australia, Department of the Attorney General, Parliamentary Counsel’s Office: How to read legislation, a beginner’s guide. 2022. Reference Source

[24] Ferolito B Valle IF do Gerlovin H : Visualizing novel connections and genetic similarities across diseases using a network-medicine based approach. *Sci. Rep.* 2022;12:14914. 10.1038/s41598-022-19244-y 36050444 PMC9436158

[25] kclquantlaw: kclquantlaw/pipeline: Sofia, an offline, cross-Act, pipeline for parsing UK’s legislation XML documents (v1.0.0). [Code]. *Zenodo.* 2023. 10.5281/zenodo.7611873

[26] kclquantlaw: kclquantlaw/graphie: Graphie: A network-based visual interface for UK’s Primary Legislation (v1.0.0). [Code]. *Zenodo.* 2023. 10.5281/zenodo.7620232 PMC10230175 37265685

