# Supplementary material for: Graphie: A network-based visual interface for the UK's primary legislation
Source: F1000Res. 2023 Mar 3;12:236. [Version 1] doi: 10.12688/f1000research.129632.1 (PMC10230175; doi:10.12688/f1000research.129632.1)
Supplement: Peer review history

# Peer review: Reviewer response for version 1

**DOI:** 10.5256/f1000research.142329.r175485

**Published:** 2023-05-30

**Authors:** Andreas Nishikawa-Pacher

**Affiliations:** 1 Department of Legal and Constitutional History, Universitat Wien, Vienna, Vienna, Austria; 2 Vienna School of International Studies, Vienna, Austria; 3 Bibliothek, Technische Universitat Wien, Vienna, Vienna, Austria

The paper presents an interesting tool that allows users to read legal texts such that cross-references (to other Acts, or to other sections of a given Act) are immediately clickable, with an optional visibility of such connections in network graphs.

 The article does a great work in embedding the project into the wider field of legal informatics and of metadata-based network graphs. Similar efforts are referenced in a way that highlights the relevance of the current project. Furthermore, the method section is detailed and offers clear clues on how the project was created. While I personally lack some of the relevant methodical skills, the section sounds plausible, replicable and comprehensible even for a semi-layman like me.

 As regards some critical words, I only have three minor issues to raise. They pertain more to the 'framing' of the text rather than the actual project per se, with the exception of the third remark on the UX of Graphie.

 First, section 3 outlines some use cases. Inter alia, "[t]hese representations could be a powerful tool for portraying the legal understanding of an ideal law practitioner that knows all the interconnections and shortcuts among different sections and Acts of the UK’s Statute Book." -- I think this is an overstatement given how law (and lawyers) operate in terms, for instance, of implicit analogies. Relevant cross-references to other legal acts (or to other legally binding documents such as court decisions) are not always made explicit verbally in legal texts. They are thus difficult to put into metadata. One of the key tasks of a lawyer's education and work is, in fact, to flesh out such *implicit *cross-references in a creative way that can hardly be detected with (semi-)automated reference-extraction, at least according to my experience. I thus would not say that Graphie portrays the understanding of an *ideal* law practitioner, but only visualizes (and facilitates) the minimum baseline of tracing cross-references.

 In other words, and to paraphrase another paper (p. 20 of an article titled 'Is Every Law for Everyone?'), an adequate visualisation of law's complexity cannot be "achieved by just dumping [... legal] statutes into [Graphie]"; or, putting legal texts into network graphs may be "a *necessary* but by no means a *sufficient* condition for" this purpose.

 Second, a few optional ideas for the Discussion section. From the authors' viewpoint, what is the ideal future for reading/accessing/visualising primary legislation? Perhaps users having a huge touch-screen operating Graphie where they read legal documents side-by-side with network graphs which they can easily open cross-Act references with a single touch? Would that make one a "better" lawyer? (Sorry for the perhaps provocative question; I am just wondering about the final "vision" of this project.) Moreover, reading the final paragraph outlining the next challenges made me think about some concrete issues, e.g., automating the insertion of modifications of laws into Graphie; or: linking legal data across different countries and other polities; or: linking primary legislation with relevant court decisions are some of these possible/impossible to achieve, and what does this tell us about the limitations of this project? Finally, are there broader implications for legal informatics? E.g., did the authors experience some kind of missing metadata that they would wish to be implemented across every single country's official legal database, or are there even already elaborate and standardized metadata schemes that official legal databases could make use of so as to better allow for a scalability of Graphie?

 The third minor aspect relates to some technicalities of Graphie itself (at least from the perspective of my own UI experience). For example, in network.html (the inbound network view), using my Chrome browser in 100% size leads the (supposedly?) "right" panel to be dropped into the bottom (or even outside) of the screen; it is only when I zoom out to 90% that I see the panel actually to the right. In addition, I find it slightly confusing that clicking on a reference leads me to the whole other section immediately, exiting the one I have been reading until then. For instance, 131(3) of the Housing Act tells me about a "final management order under section 102(8)", with some more text following after that reference, but if I seek to have a quick view of the referenced section 102(8), a click on it forces me to exit the paragraph that I have not even finished reading yet, leading me to section 102 without highlighting my relevant sub-part nr. 8 which is somewhere in the bottom of the browser, requiring me to scroll down a while, which I would only do if I actively read & memorized the correct paragraph number, which, in turn, I believe I should actually not do if the tool was to offer me an "easier" reading experience. In that way, the (assumed) task of smoothing the cross-reading of sections is not achieved in the optimal way, but the tool rather generates new cognitive burdens that one must take into account. While reading 131(3), perhaps the opening of a tiny box showing (a preview of?) 102(8) would be better. These are just very minor technical issues that in no way lessen the quality of the paper. The deeper implication is that a validation of the UI experience, or at least a discussion thereof, would be great.

 Overall, the article is excellent, the tool innovative & timely; my comments simply address three minor issues.

Are the conclusions about the tool and its performance adequately supported by the findings presented in the article?

Partly

Is the rationale for developing the new software tool clearly explained?

Yes

Is the description of the software tool technically sound?

Yes

Are sufficient details of the code, methods and analysis (if applicable) provided to allow replication of the software development and its use by others?

Yes

Is sufficient information provided to allow interpretation of the expected output datasets and any results generated using the tool?

Partly

Reviewer Expertise:

Relevant for this paper: (a) general understanding of law and legal databases / legal informatics; (b) meta-science, including the data quality of metadata about scientific outputs and the role of APIs

I confirm that I have read this submission and believe that I have an appropriate level of expertise to confirm that it is of an acceptable scientific standard.

## References

[1] : Is Every Law for Everyone? Assessing Access to National Legislation through Official Legal Databases around the World. *Oxford Journal of Legal Studies* .2023; 10.1093/ojls/gqac032 10.1093/ojls/gqac032 PMC1024392837287901

# Peer review: Reviewer response for version 1

**DOI:** 10.5256/f1000research.142329.r172963

**Published:** 2023-05-25

**Authors:** Nicola Lettieri

**Affiliations:** 1 INAPP - National Institute for Public Policy Analysis, Rome, Italy; 2 Department of Law, Universita degli Studi del Sannio, Benevento, Campania, Italy; 3 Department of Law, Libera Universita Internazionale degli Studi Sociali Guido Carli, Rome, Lazio, Italy

The paper presents Graphie, a tool implementing a network-based visual interface that allows exploring UK primary legislation in an intuitive and user-friendly way.

 After briefly introducing the rationale behind the research, the authors describe the system's building blocks, all gathered within an offline data pipeline that includes parsing, data modelling and visualisation functionalities.

 The work is undoubtedly timely: the application of network analysis and visualisation techniques to legal corpora is a topic that, in recent years, has drawn the attention of different disciplinary fields (from law to computational legal studies) that still communicate relatively little. 

 The text is well-written, the structure is coherent, and the arguments are consistent. One relief can be made about the level of novelty of the proposal, a profile that is not sufficiently addressed in the related works section. Authors do not consider a series of papers that have already dealt with the application of network analysis and visualization techniques to complex legal corpora encompassing not only legislation and statutes but also legal literature, preliminary works and case law. 

 For the sake of completeness of inquiry, we draw authors' attention to several papers that have touched precisely on this type of application not only to enable more advanced and intuitive forms of access to legal information but also to enable a deeper understanding of the evolution of legal systems.

 The authors could consider such a circumstance to better argue in favour of the originality of their work. Among the works closest to the topics the paper deals with:

- Lettieri, N., Altamura, A., & Malandrino, D. (2017). The legal macroscope: Experimenting with visual legal analytics. Information Visualization, 16(4), 332-345. https://doi.org/10.1177/1473871616681374 
- La Cava, L., Simeri, A., & Tagarelli, A. (2022, July). LawNet-Viz: A Web-based System to Visually Explore Networks of Law Article References. In Proceedings of the 45th International ACM SIGIR Conference on Research and Development in Information Retrieval (pp. 3300-3305). - https://doi.org/10.1145/3477495.3531668
- Lettieri, N., Altamura, A., Faggiano, A., & Malandrino, D. (2016). A computational approach for the experimental study of EU case law: analysis and implementation. Social Network Analysis and Mining, 6, 1-17. https://doi.org/10.1007/s13278-016-0365-6 
- Moodley, K., Hernandez-Serrano, P. V., Zaveri, A. J., Schaper, M. G., Dumontier, M., & Van Dijck, G. (2020). The case for a linked data research engine for legal scholars. European Journal of Risk Regulation, 11(1), 70-93. - doi:10.1017/err.2019.51 . 
- Górski, Ł. (2021). Network Science in Law: A Framework for Polish Case-Law Citation Network Analysis. IT Professional, 23(5), 62-66. https://doi.org/10.1109/MITP.2020.3037354

Are the conclusions about the tool and its performance adequately supported by the findings presented in the article?

Yes

Is the rationale for developing the new software tool clearly explained?

Yes

Is the description of the software tool technically sound?

Yes

Are sufficient details of the code, methods and analysis (if applicable) provided to allow replication of the software development and its use by others?

Yes

Is sufficient information provided to allow interpretation of the expected output datasets and any results generated using the tool?

Yes

Reviewer Expertise:

Computational legal studies, law and complexity

I confirm that I have read this submission and believe that I have an appropriate level of expertise to confirm that it is of an acceptable scientific standard.

## References

[1] : The legal macroscope: Experimenting with visual legal analytics. *Information Visualization* .2017;16(4) : 10.1177/1473871616681374 332-345 10.1177/1473871616681374

[2] : LawNet-Viz: A Web-based System to Visually Explore Networks of Law Article References. *SIGIR '22: Proceedings of the 45th International ACM SIGIR Conference on Research and Development in Information Retrieval* .2022; 10.1145/3477495.3531668 3300-3305 10.1145/3477495.3531668 Reference source

[3] : A computational approach for the experimental study of EU case law: analysis and implementation. *Social Network Analysis and Mining* .2016;6(1) : 10.1007/s13278-016-0365-6 10.1007/s13278-016-0365-6

[4] : The Case for a Linked Data Research Engine for Legal Scholars. *European Journal of Risk Regulation* .2020;11(1) : 10.1017/err.2019.51 70-93 10.1017/err.2019.51

[5] : Network Science in Law: A Framework for Polish Case-Law Citation Network Analysis. *IT Professional* .2021;23(5) : 10.1109/MITP.2020.3037354 62-66 10.1109/MITP.2020.3037354
